# Diversity and distribution of eukaryotic microbes in and around a brine pool adjacent to the Thuwal cold seeps in the Red Sea

**DOI:** 10.3389/fmicb.2014.00037

**Published:** 2014-02-04

**Authors:** Yong Wang, Wei Peng Zhang, Hui Luo Cao, Chun Shum Shek, Ren Mao Tian, Yue Him Wong, Zenon Batang, Abdulaziz Al-Suwailem, Pei-Yuan Qian

**Affiliations:** ^1^Division of Life Science, Hong Kong University of Science and TechnologyHong Kong, China; ^2^Sanya Institute of Deep Sea Science and Engineering, Chinese Academy of SciencesSan Ya, China; ^3^Coastal and Marine Resources Core Laboratory, King Abdullah University of Science and TechnologyJeddah, Saudi Arabia

**Keywords:** Eukaryotic microbes, 18S rRNA, cold seep, brine pool, classification

## Abstract

A hypoxic/suboxic brine pool at a depth of about 850 m was discovered near the Thuwal cold seeps in the Red Sea. Filled with high concentrations of hydrogen sulfide and ammonia, such a brine pool might limit the spread of eukaryotic organisms. Here, we compared the communities of the eukaryotic microbes in a microbial mat, sediments and water samples distributed in 7 sites within and adjacent to the brine pool. Taxonomic classification of the pyrosequenced 18S rRNA amplicon reads showed that fungi highly similar to the species identified along the Arabic coast were almost ubiquitous in the water and sediment samples, supporting their wide distribution in various environments. The microbial mat displayed the highest species diversity and contained grazers and a considerable percentage of unclassified species. Phylogeny-based methods revealed novel lineages representing a majority of the reads from the interface between the sea water and brine pool. Phylogenetic relationships with more reference sequences suggest that the lineages were affiliated with novel Alveolata and Euglenozoa inhabiting the interface where chemosynthetic prokaryotes are highly proliferative due to the strong chemocline and halocline. The brine sediments harbored abundant species highly similar to invertebrate gregarine parasites identified in different oxygen-depleted sediments. Therefore, the present findings support the uniqueness of some microbial eukaryotic groups in this cold seep brine system.

## Introduction

Eukaryotic microbes are important players in marine environments. Depending on the presence of organic debris and prokaryotic cells, heterotrophic eukaryotes dominate the deep sea ecosystems and tend to spread in the direction of nutrient source (Caron et al., 1999; Schaechter, 2012). Fungi are important degraders that can utilize various carbon sources and widely distributed on the marine subsurface (Edgcomb et al., 2011a). The diversity of fungal species decreased from shallow to deep-sea sediments (Edgcomb et al., 2011a). But a recent study provides evidence of higher fungal species richness in subseafloor than previously estimated (Orsi et al., 2013). On the other hand, eukaryotic microbes are also grazers that control the populations of prokaryotes in the hydrothermal vents and cold seeps. For instance, ciliates (Alveolata) actively grazed on microbial mats adjacent to cold seeps (Takishita et al., 2010). Eukaryotic inhabitants in anoxic biospheres are highly represented by gregarine parasites (Excavata) present in the animal gut (Morrison et al., 2007; Hampl et al., 2009). Other eukaryotes such as ciliates have developed symbiotic relationships with archaea and bacteria while adapting to anoxic environments (Van Hoek et al., 2000; Orsi et al., 2012a). Nevertheless, a free-living anaerobic Excavata species, which is a grazer of bacteria, was discovered in anoxic coral-reef sediment (Ekebom et al., 1996). However, the strategies that these eukaryotic microbes developed to achieve fitness may, on the other hand, affect the scope and mode of their spread in different ecosystems.

Eukaryotic microbial communities in some extreme environments have been investigated in recent years. Such a progress enables us to compare the communities from different locations and environments, and to further postulate the spreading mode of the eukaryotic microbes in the oceans. In hypersaline deep-sea basin in the Mediterranean Sea, protist communities along water columns and brine-sea water interface were investigated, which resulted in identification of novel kinetoplastids (Edgcomb et al., 2011c). Unique protist lineages were also identified in a brine pool near an offshore station (Edgcomb et al., 2014). In abyssal plains, the novel Alveolata and Euglenozoa species (closely related to parasites) dominated the 18S rRNA clones isolated from southeastern Atlantic sites with a depth >5000 m (Scheckenbach et al., 2010). The high diversity of the eukaryotic species present in other abyssal areas was confirmed in another study, which demonstrated that marine sediments are a reservoir of eukaryotic DNA derived from the overlying water body (Pawlowski et al., 2011). In these open benthic locations, water circulation can mix different populations that are geographically separated. However, in isolated and rare environments, the selective pressure of the extreme conditions drives the evolution of populations attempting to colonize the new niche. For example, in the Black Sea, the oxic–anoxic transition zone supports a large number of chemoautotrophic prokaryotes (Lam et al., 2007; Glaubitz et al., 2010) that in turn, attract eukaryotic grazers and other extremophilic species. A recent study of eukaryotic communities in the Black Sea revealed a global distribution of eukaryotic microbial species, as well as a great diversity of unclassified ciliates and stramenopiles with unknown ecological functions in sulfidic and anoxic zones (Wylezich and Jürgens, 2011). These studies suggest that the eukaryotic species were substantially specialized to the environments of the ecological niches. The development of environmental stress-resistant and nutrient-uptake capacities will facilitate spreading in the new locations.

In the Red Sea, cold seeps and a brine pool were recently discovered in Thuwal basin with a depth of 850 m (Batang et al., 2012). The environment at the bottom of the pool has a high sulfide concentration, and the seepage of methane had been terminated as indicated by the extinction of methane-consuming mussels around the seeps (Batang et al., 2012). A systematic study of eukaryotic microbial communities will help to understand a developed model ecosystem for cold seeps. Although eukaryotic microbes in cold seep ecosystems have been investigated in several previous studies (Bernhard et al., 2001; Edgcomb et al., 2007; Takishita et al., 2010; Nagahama et al., 2011), a comprehensive analysis of the waters and sediments from different seepage sites has not been conducted. In the present study, we employed 18S rRNA pyrosequencing to study the eukaryotic communities present in bottom water, brine water, a microbial mat and sediments in the Thuwal seep system and provide evidence for the uniqueness and distribution of eukaryotic species around the cold seeps.

## Materials and methods

### Samplings

The Thuwal cold seeps (22°16N–38°53E) were discovered by *R/V Aegaeo* in 2011 during a KAUST Red Sea exploration cruise (Batang et al., 2012). Sampling was conducted by a remotely operated vehicle (ROV *Max Rover*, DSSI, USA) around a brine pool (2.2 km^2^ area) that approximated seeping vents at a depth of about 850 m. The temperature, salinity and concentrations of dissolved oxygen and methane were measured from the sea surface downwards to the brine pool by the equipped METS and CTD. The ROV was controlled onboard to obtain sediment push cores from 2 sites outside of the brine pool and 2 within the pool; a microbial mat sample on the bank of the pool; water samples above the seafloor; and from the interface water between the pool and the bottom water using 4 1-L Niskin bottles (Table 1). The water samples were filtered through a 0.22-μm polycarbonate membrane (47-mm diameter, Millipore, Massachusetts, UK). The polycarbonate membrane was then frozen in 0.8 mL of DNA extraction buffer. The concentrations of total organic carbon (TOC) and total nitrogen (TN) were determined using a TOC/TN analyzer in the laboratory (TOC-VCPH/TNM-I, Simadzu, Kyoto, Japan). Ammonia concentration was measured with a Vis/UV spectrophotometer (PerkinElmer, Waltham, MA, US) (Koroleff, 1970). For the sediments and microbial mat, pore waters were collected to perform measurements.

**Table 1:** **Environmental conditions of the sampling sites**.

**Sample**	**ID**	**Coordinate**	**TOC(mg/L)**	**TN(mg/L)**	**O_2_(%)**	**Salinity(‰)**
Top of mat	Mat	22°16.999N- 38°53.893E	35.95	7.87	25	43
Bottom water	NDW	22°17.100N- 38°52.90E	19.22	2.6	25	43
Interface water	DBI	22°17.2767N- 38°53.473E	23.58	6.32	~7.8	60
Bottom sediment	DS3	22°17.100N- 38°53.075E	76.38	12.49	24.4	43
Bottom sediment	DS6	22°17.100N- 38°52.90E	71.04	5.48	25	43
Brine sediment	BS8	22°17.210N- 38°53.736E	60.88	17.13	~0.5	96
Brine sediment	BS9	22°17.313N- 38°53.645E	58	16.87	0.2	96

The brine pool was located at a depth of about 850 m near the Thuwal cold seeps. The interface water was sampled between the normal sea water and brine pool. The brine sediments were collected from the bottom of the brine pool; the bottom sediments were referred to the surface sediments of the seafloor around the pool; the microbial mat sample was obtained from surface of a sediment core collected near one of the cold seeps.

### Pyrosequencing of barcoded 18S rRNA gene amplicons

Total genomic DNA was extracted from 10 g of surface sediments and the microbial mat using the PowerSoil®DNA Isolation Kit (MO-BIO, USA) according to the manufacturer's instructions and from water samples according to the modified SDS-based method described by Lee et al. (2009). Subsequently, a purification step was carried out using the MoBio Soil DNA Isolation Kit (MoBio Laboratories, Inc., Carlsbad, CA, USA). The quality and quantity of the DNA were determined using a NanoDrop spectrophotometer (ND-1000, NanoDrop, USA) and by gel electrophoresis. Purified DNA samples were maintained at −20°C for future use.

The different samples were amplified by PCR using primers with different barcodes. A set of primers was designed by adding an 8-nucleotide (nt) barcode (Table 2) to the universal forward primer 1A (5′- AACCTGGTTGATCCTGCCAG-3′) (Medlin et al., 1988) and reverse primer 564R (5′- GGCACCAGACTTGCCCTC-3′) (Amann et al., 1990), targeting the hypervariable regions V1–V3 of the 18S rRNA gene. A 25-μL PCR reaction consisted of 1U of *Pfu* Turbo DNA polymerase (Stratagene, La Jolla, CA, USA), 1 × *Pfu* reaction buffer, 0.2 mM dNTPs (TaKaRa, Dalian, China), 0.1 μM of each barcoded primer, and 20 ng of genomic DNA template. The PCR reaction was performed on a thermocycler (MJ Research Inc., Bio-Rad, USA) with an initial denaturation at 94°C for 5 min, followed by 25 cycles of 94°C for 50 s, 59°C for 50 s, and 72°C for 50 s, and a final extension at 72°C for 5 min. The PCR products were purified using the TaKaRa Agarose Gel DNA Purification Kit (TaKaRa, Dalian, China) and quantified using a NanoDrop device. A mixture of the PCR products was prepared and then pyrosequenced using the ROCHE 454 FLX Titanium platform.

**Table 2:** **Diversity and coverage of the 18S rRNA amplicon reads at a dissimilarity of 3%**.

**Sample ID**	**Barcode**	**Eukaryotic reads**	**No. OTUs**	**Shannon index**	**Chao1**	**Good's coverage**
Mat	CGGATTGC	1060	327	6.72	923	0.305
NDW	ATGCAGTC	13,074	177	4.33	199	0.781
DBI	ATATTCGC	4569	305	3.84	536	0.487
DS3	TTATCCGC	1261	42	1.53	72	0.419
DS6	TATCGTCC	4681	56	1.20	131	0.281
BS8	ACCACAAC	9565	111	1.28	263	0.357
BS9	TCTGCGTT	163	27	2.80	37	0.5

Sample IDs were described in Table 1.

### Calculation of species richness and taxonomic assignment of pyrosequencing reads

The pyrosequencing data were deposited in the NCBI Sequence Read Archive (SRA) database under the accession number SRP031839. A subsequent bioinformatics analysis was performed using the QIIME package version 1.3.0 (Caporaso et al., 2010b). Raw reads with the following characteristics were filtered out: an average flowgram score of <25, a quality window of <50 bp, ≥1 ambiguous nucleotides, <150 bp, and containing homopolymers of >6 bp or without a complete barcode. Qualified reads were assigned to the corresponding samples according to the barcodes and then subjected to denoising using Denoiser (Reeder and Knight, 2011). Chimeric reads were identified using ChimeraSlayer (Haas et al., 2011) and then removed. Taxonomic assignment of qualified reads was conducted using the RDP classifier (Cole et al., 2009) against Silva108 with a bootstrap confidence level of 50%. Archaeal reads were removed from the data, and the remaining eukaryotic reads were clustered using UCLUST and then assigned to operational taxonomic units (OTUs) at a 97% similarity. The representative reads, which were most abundant in each OTU, were selected for alignment using PyNAST (Caporaso et al., 2010a) against the Silva108 database (Quast et al., 2013). Species diversity and richness were computed at 97% similarity as a part of the QIIME's beta diversity pipeline. Three metrics were calculated including observed species, Chao1 (a species richness index), and Shannon index (a diversity index that takes into account both abundance and evenness) after rarefying the samples at the smallest-sized library (Lemos et al., 2011). The Good's coverage was also calculated at the same similarity (Good, 1953).

### Elimination of reads containing false positive amplicons

Unclassified eukaryotic reads were randomly selected for NCBI BLAST search. Some of them were highly similar to protein-coding genes. The presence of unclassified eukaryotic reads was probably due to false positive PCR amplification of non-rRNA genes. To remove these reads, representative reads from OTUs at a similarity of 3% were selected and screened for the presence of other 18S probes. Between the target regions 1A and 564R, there were 2 eukaryotic 18S rRNA probes: Euk381 (5′-TCCGGAGAGGGAGCC-3′) and Euk422 (5′- GGCAGCAGGCACGAA-3′) (Medlin et al., 1988). The 18S rRNA reads should have a complete primer sequence and at least one of the probes. When the probes were aligned with the reads, 2 mismatches were allowed. The algorithm used for alignment has been introduced in our previous work (Wang and Qian, 2009).

### *DE NOVO* classification of 18S rRNA amplicon reads using a phylogenetic method

For unclassified and environmental eukaryotes, an alternative taxonomic classification of eukaryotic reads was developed to determine their positions in the eukaryotic kingdoms. We adopted the standard for eukaryotic taxonomy as listed in SILVA version114 (Quast et al., 2013). The protocol was based on the reconstruction of phylogenetic relationships between the 18S amplicons reads and reference sequences from different eukaryotic kingdoms. Representative reads for the eukaryotic OTUs at a dissimilarity of 20% were retrieved, and 47 singleton OTUs with one read in all the samples were removed. The reference sequences were downloaded from the SILVA database. New emerging groups, such as Zeuk77 and SA1-3C06, were also included in the dataset, resulting in a collection of reference sequences from a total of 23 kingdoms (or equivalents). Five representative 18S rDNA sequences were obtained from different taxonomic sublevels of each kingdom.

Multiple alignment of the combined dataset was conducted using MUSCLE3.5 (Edgar, 2004). The alignment downstream of the end of the reads was trimmed. After a manual assessment of the alignment, 14 OTUs were deleted from the dataset. As a result, 98 representative reads (average size: 525 bp; GenBank accession number: KJ003881-KJ003980) for the OTUs and 103 reference eukaryotic references were used to reconstruct a Bayesian phylogenetic tree. We used Jmodeltest2.1.3 (Darriba et al., 2012) to select the best substitution model for the sequences. BEAST1.7.5 (Drummond and Rambaut, 2007) was used to perform the Bayesian phylogenetic analysis using the substitution model HKY+G+I, as suggested by the results obtained using the Jmodeltest. The first 20% of the 10000 trees were discarded, and the rest were summarized. The final results were visualized in iTOL (Letunic and Bork, 2011), which clustered the sequences into triangle clades. On each clade, reference kingdoms were identified (if available), and the percentage of reads in the given clade was calculated for the 7 cold seep samples. The distribution of the reads for the 98 OTUs in different clades was used to calculate Bray-Curtis dissimilarity between the samples, which was then used as an input for principal component analysis (PCA).

To display the intra-structures of clades 2, 3, and 5 in Figure 2, additional sequences were collected from the BLASTN results. The alignments of these sequences were refined using MUSCLE3.5 and evaluated manually. MEGA5 was used to reconstruct neighbor-joining (NJ) trees with 1000 bootstrap replicates (Tamura et al., 2011); consensus MJ phylogenetic trees were then obtained based on 1000 bootstrap replicates using the NJ tree as a reference. Outgroup sequences for the ML trees were selected from the neighboring clades in Figure 2.

### Isolation and identification of fungal species

To cultivate fungi in the brine sediments, samples from the surface of BS8 were diluted with 35 ppt autoclaved seawater and then inoculated onto agar plates using a medium for fungi (20 g potato dextrose agar, 35 g sea salt, 8 g agarose, and 1 L distilled water). After cultivation at 28°C for 5 days, pure colonies of the fungi were isolated in the above medium. Next, different fungal colonies were identified based on 18S rRNA sequences. Thus, the genomic DNA of isolated fungal colonies was extracted using the PowerSoil® DNA Isolation Kit (MO-BIO, USA) according to the manufacturer's instructions. The 18S rRNA sequences were PCR-amplified using primers U564F (5′- GTGYCAGCMGCCGCGGTAA - 3′) and U1390R (5′- TTGYACACACCGCCCGTC -3′). The PCR reaction mixture contained 1.25 U of Taq DNA polymerase (New England Biolabs, England), 2 μ L 10× PCR buffer (15 mM Mg^2+^), 1 μ L of dNTPs (2.5 mM), 2 μ L (~30 ng) DNA template, and 0.5 μ L of each primer (10 μ M) to achieve a final volume of 20 μ L. PCR was performed using the following cycle: initial denaturation at 94°C for 5 min; 28 cycles at 94°C for 1 min, 50°C for 1 min, 72°C for 1 min; and a final extension at 72°C for 10 min. The PCR products were sequenced and resolved using NCBI BLAST.

## Results

### Environmental conditions at the sampling sites

Seven samples were collected from the Thuwal cold seep (Table 1). The marine sediment sample DS3 was obtained from the bank of the brine pool; sediment DS6 was collected from a location further away from the brine pool. Hypersaline marine sediments BS8 and BS9 were collected within the brine pool, but the BS8 sampling site was shallower and closer to the bank and seeps. The bottom of the brine pool was an oxygen minimum zone, with a measured oxygen concentration of close to zero (Table 1). The salinity reached 96‰ in the pore water of BS8 and BS9. The concentration of hydrogen sulfide in the almost anoxic brine pool was >200 μMol/L and the concentration of ammonia at sites BS8 and BS9 was 12.7 and 13.9 mg/L, respectively. In the interface between the normal bottom water and the brine water (DBI), dissolved oxygen was approximately 8% and salinity was 60‰. The temperature of the 7 sites was similar at approximately 22°C. The methane detector did not reveal notable differences in methane concentration from the surface down to the sea floor along the water column. The TOC was more abundant in the sediment pore waters than in the water samples and microbial mat. Amongst the sediments, bottom sediments DS3 and DS6 had a higher amount of TOC but a lower amount of TN (Table 1).

### Similarity-based classification and diversity of 18S amplicon reads

Archaeal reads were filtered from the pyrosequenced 18S rRNA amplicons prior to calculating the diversity of the eukaryotic microorganisms. The results showed that the microbial mat sample (Mat) ranked highest with respect to the 2 diversity indexes Shannon and Chao1 at 3% dissimilarity (Table 2). The eukaryotic microbial communities in the water samples (normal sea water above the sea bottom [NDW] and DBI) were more diverse than those in the sediments if only the Shannon index was considered. A Good's coverage for NDW was only 0.78 and ranked first among all samples, although 13,074 reads were obtained. Thus, the coverage values indicated that the pyrosequencing depth might not be sufficient to cover most of the species.

**Figure 1:**
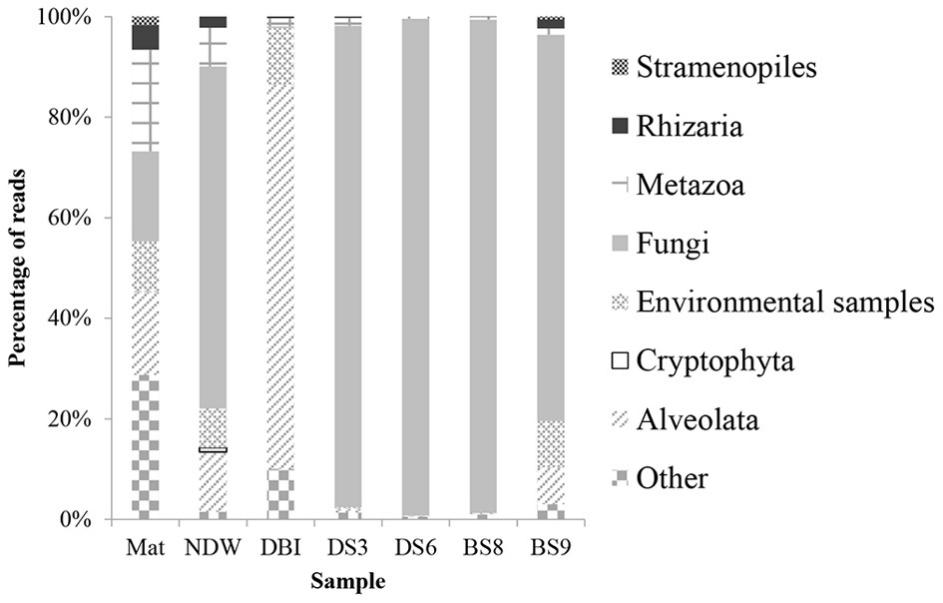
**Taxonomic classification of eukaryotic 18S amplicon reads using the QIIME pipeline**. The qualified 18S rRNA amplicon reads were classified into kingdoms using the RDP classifier against Silva108 with a confidence level of 50%. The sample IDs were defined in Table 1.

Classification of the amplicon reads against the SILVA database revealed 6 kingdoms or subkingdoms in the 7 samples (Figure 1). The NDW and the 4 sediment samples were predominately fungi, which ranged from 68 to 99% of the eukaryotic reads. In particular, >95% of the reads were sorted to fungal species in DS3, DS6, and BS8. Almost all of the fungal reads belonged to the *Acremonium* genus. The DBI differed from the other samples by sustaining a high proportion of Alveolata (76%). In contrast, Mat contained 3 dominant phyla, which were attributed to its high level of species diversity. Metazoa were particularly enriched in Mat, as Nematoda (3.5%) and Annelida (2.1%) could be identified in the microbial mat. However, 13% of the metazoan inhabitants could be unclassified to lower levels. Unclassified and environmental eukaryotic species also accounted for a considerable percentage of the reads in NDW, DBI, and BS9, which ranged between 9% and 39% of the entire community (Figure 1).

### Phylogeny-based position of unclassified eukaryotic species

**Figure 2:**
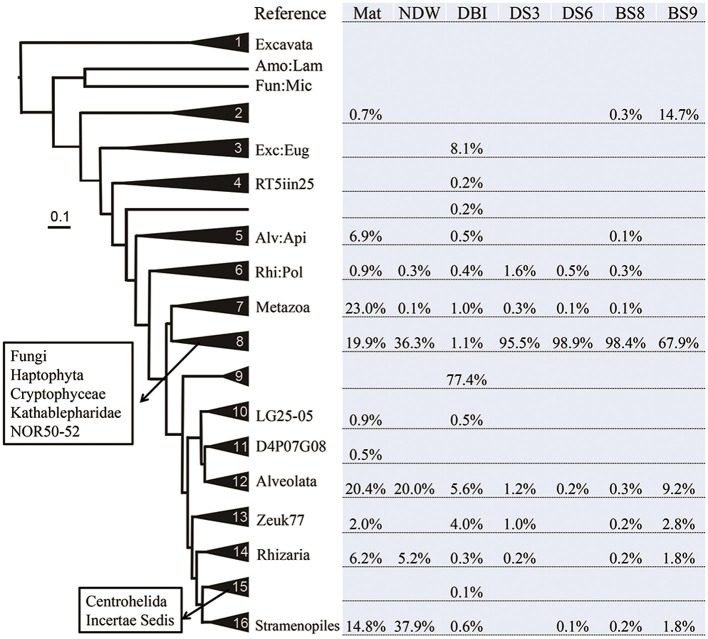
**Distribution of eukaryotic microbial communities in different clades of the Bayesian phylogenetic tree**. The reference eukaryotic groups are labeled in reference to their 18S sequences. The sample IDs are defined in Table 1. Lamproderma from Amoebozoa is abbreviated Amo:Lam; Microsporidia from Fungi is abbreviated Fun:Mic. Euglenozoa, Apicomplexa, and Polycystinea are shortened in the same manner. RT5iin25, NOR50-52, LG25-05, D4P07G08, and Zeuk77 are new eukaryotic groups that were collected by SILVA database in version 114.

Considering the high percentage of unclassified eukaryotic reads in some samples, phylogeny-based methods were employed to determine the evolutionary positions of these potentially novel lineages. Representative reads from OTUs at 20% dissimilarity were selected from a new set of highly qualified reads from which all potentially false-positive amplicons and chimeric reads had been filtered out (Table 1). The representative reads and reference 18S sequences from the different kingdoms were used to generate a Bayesian phylogenetic relationship. The tree was simplified to maintain all sequences in the 16 clades (each comprising at least 3 sequences) and 3 long branches (Figure 2). The relationships were well supported by a Bayesian posterior probability of >0.5, excluding the divergence between clades 4–6. The distribution of the amplicon reads in different clades is presented as a percentage. Most of the reads (92%) from Mat were affiliated with the super kingdoms SAR (Alveolata, Stramenopiles and Rhizaria) and Opisthokonta (Fungi and Metazoa). These species in Mat were somewhat distantly related to the currently known SAR and Opisthokonta organisms, because only 61% of the reads could be sorted into known eukaryotic kingdoms by QIIME. The dominant metazoans nourished by the microbial mat were probably nematodes (95% similar to *Viscosia viscosa* based on BLAST results of 18S rRNA sequences). Following the nematodes, Annelida species (95% similar to *Aurospio dibranchiate*) also populated on the mat. All together, the metazoans accounted for 23% of the eukaryotes in Mat. As mentioned above, fungi dominated the sediment samples, but their percentages in NDW and BS9 were lower than those generated by QIIME. Stramenopiles was the most abundant kingdom in NDW, and Alveolata accounted for more qualified reads in NDW and BS9. This finding might be explained by the affiliation of some environmental and unclassified reads in the results of QIIME. Three OTUs in clade 9 formed an independent group between SAR and Opisthokonta, which accounted for approximately 77% of the amplicon reads for DBI. However, they were classified as Alveolata by the QIIME pipeline.

Clades 1–5 in Figure 1 exhibited a large distance from the rest of the tree, and they comprised reads from Mat, DBI and BS9, likely due to a long-branch attraction (LBA) phylogenetic effect (Hampl et al., 2009). Clade 1 includes the basal-derived Excavata organisms (Hampl et al., 2009). The 2 sequences from Lamproderma of Amoebozoa and Microsporidia of Fungi intersected the long branch between clades 1 and 2, and both have been reported to show a discrepancy between traditional and 18S rRNA-based phylogenetic classification methods (Dawson and Pace, 2002; Nagahama et al., 2011; Fiore-Donno et al., 2012). Clade 2 did not posit with a reference sequence; the reads from BS9 were most frequently detected in this clade. A minority of the reads in this clade were from Mat and BS8. A sequence derived from Euglenozoa *Bodo saltans* served as the basal sequence in clade 3, which suggested that 8.1% of the eukaryotic species in the DBI originated from the Euglenozoa.

Several reference groups were listed in the SILVA database as emerging eukaryotic groups because of their greater or lesser divergence from known kingdoms. Some of them, including RT5iin25, NOR50-52, LG25-05, D4P07G08, and Zeuk77, were unambiguously positioned in the phylogenetic tree and did not cluster with known kingdoms (Figure 2). The sequences from these reference groups were similar to the reads obtained from the cold-seep samples. For example, Zeuk77-related species were distributed in all samples excluding NDW and DS6.

**Figure 3:**
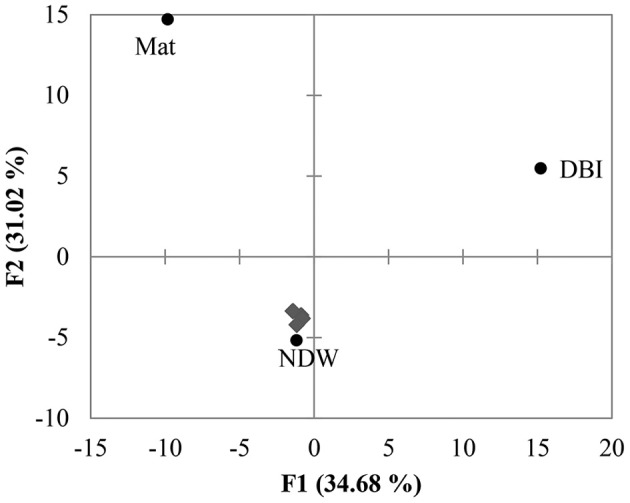
**Principal component analysis (PCA) of OTUs**. The 18S amplicon reads were clustered into OTUs at a dissimilarity of 20%. As shown in Figure 2, the distribution of the reads in the clades was used to perform PCA. The 4 sediment samples (gray diamonds) were grouped with the normal sea water above the seafloor (NDW).

PCA was performed using the distribution of reads in different clades shown in Figure 2. The results revealed a large distance between Mat, DBI and the other 5 samples (Figure 3). The first and second principals accounted for 65% of the variations. The NDW could not be separated easily from the sediment samples, which resulted in 3 distinctive groups in the cold seep system.

### Intra-phylogenetic structures of clades 2, 3, and 9

Because clades 2, 3, and 9 had only one reference sequence, we were interested in their fine-scale structure and the environmental characteristics of the additional members of these clades. BLAST searches of the NCBI-nr database were conducted to obtain additional reference sequences. Consistent with the position of clade 2 in Figure 2, the reads from the cold-seep samples were distantly related to Amoebozoa represented by *Lamproderma puncticulatum* and *Entamoeba histolytica* (Figure S1). Known species in this clade were crustacean and amphipod gregarine parasites (Prokopowicz et al., 2010; Rueckert et al., 2011) belonging to the Apicomplexa of the Alveolata kingdom. The sequences from the uncultured eukaryotes were obtained from oxygen minimum zones from a variety of locations worldwide, such as Peru Margin, the Arabian Sea and the Gulf of Maine. However, OTUs of Mat-847, and DS6-105 were present in oxic bottom sites, and therefore, the species in clade 2 are maybe not an obligate anaerobe. The largest OTU was BS9-304, and their closest relatives were detected in cold seep sediments of Sagami Bay in Japan.

**Figure 4:**
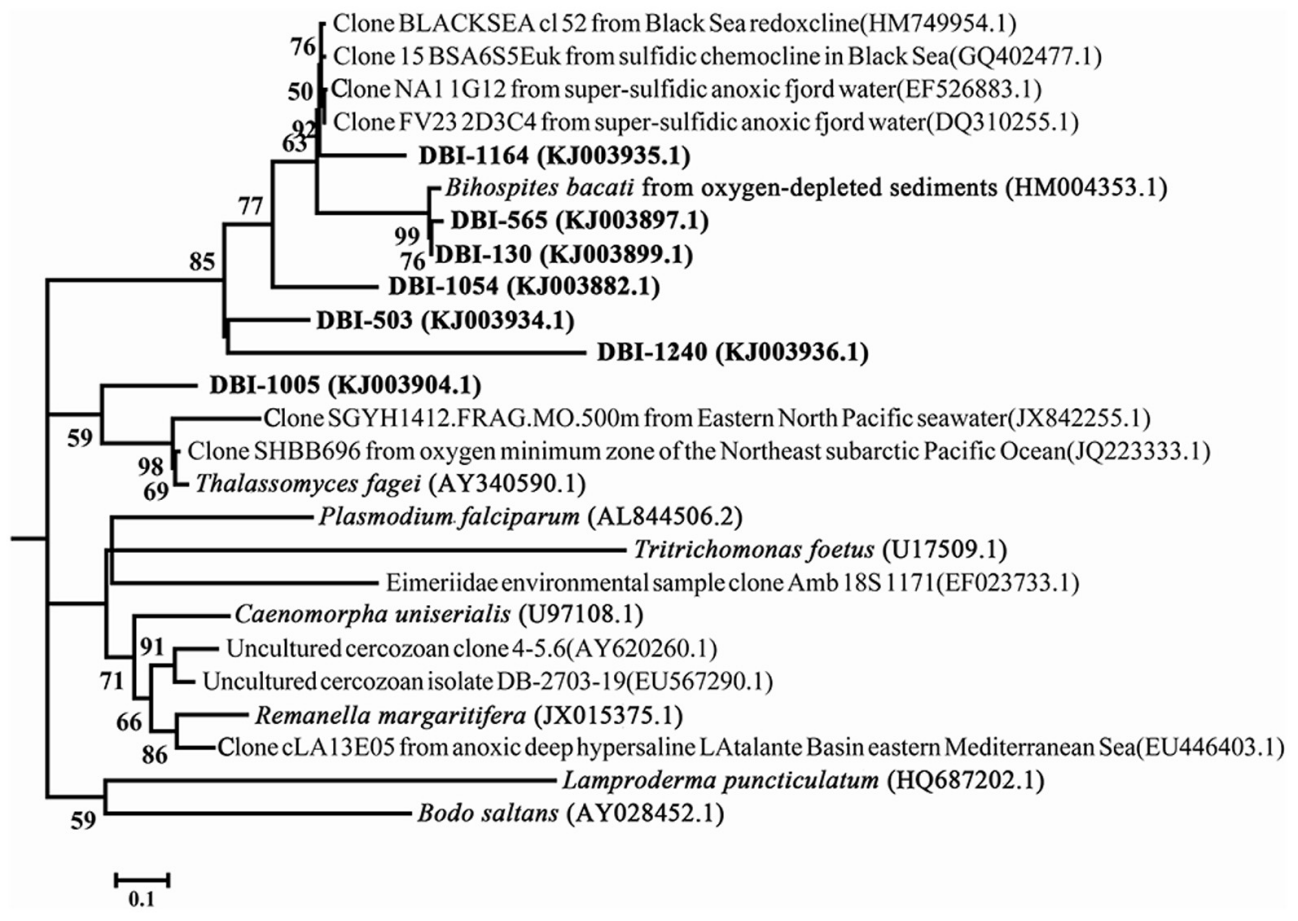
**Intra-structure of clade 3 with more reference sequences**. The 7 OTUs selected at a dissimilarity of 20% constitute clade 3 in Figure 2. The only reference sequence in the clade was from *Bodo saltans*. Bootstrap values >50 in the maximum-likelihood phylogenetic tree are shown.

Clade 3 in Figure 2 consisted of mostly reads from DBI because there was only one reference sequence from Euglenozoa. When the fine-scale structure was displayed using a maximum-likelihood (ML) tree for clade 3, 4 independent clusters could be discerned (Figure 4). Excluding DBI-1005, 6 other selected DBI OTUs were grouped together in one cluster with a *Bihospites bacati* (Euglenozoa) 18S sequence and several sequences from anoxic, sulfidic marine sediments. The most abundant OTU was DBI-130, which had 273 reads (6.6% of all reads in DBI); its representative read was tightly associated with the *B. bacati* species identified in oxygen-depleted sediment. A small OTU DBI-1164 was highly similar to cloned sequences obtained from anoxic fjords and the Black Sea. The OTU DBI-1240, consisting of 4 reads, displayed a large distance compared to the others. Thus, among the environmental sequences collected for clade 3, there were no relatives determined for 4 OTUs, and the positions of these OTUs appeared to be closer to the root of Euglenozoa. The reference species in the other 3 clusters comprised the Alveolata parasites.

Clade 9 harbored the largest eukaryotic group in DBI, and almost all of the reads in this clade sorted into the DBI-298 OTU. Two OTUs, DBI-164 and DBI-236, were much smaller, accounting for approximately 1% of the read numbers for DBI-298. The 3 OTUs stand out from the main cluster and showed a large genetic distance compared to the other reference sequences (Figure S2). This long branch was supported by high bootstrap values. The taxonomy of the closely associated sequences in the ML tree indicated that the 3 OTUs originated from the Alveolata. Another cluster of the ML tree comprised *Pyrenomonas salina* and *Acanthamoeba* sp. belonging to Cryptophyta and Amoebozoa, respectively.

### Culturable fungi from the brine sediment

Using the sediment of BS8, fungal colonies were successfully developed under laboratory conditions on standard culture medium for fungi cultivation. The 18S rRNA genes of 4 colonies were cloned and sequenced. The results of the BLAST searches revealed that they were 99% similar or identical to the *Sarocladium strictum* and *Acremonium* sp. identified on the Arabic seafloor (Jebaraj et al., 2010). QIIME pipeline sorted >95% of the pyrosequenced reads into these fungal species. The results suggest that most of the eukaryotic species in BS8 belonged to the Ascomycota fungi.

## Discussion

In the present study, we investigated the distribution and diversity of eukaryotic microbes around the Thuwal cold seeps using pyrosequenced 18S amplicons. Most of the reads from the bottom waters and sediments could be sorted into known phyla and species, and they were largely composed of culturable fungi together with some metazoans. A recent work investigated phylogenetic relationships between kingdoms by combining the informative sites in small and large ribosomal RNAs (Marande et al., 2009). In the current study, we used 454 pyrosequencing reads with a maximum size of 600 bp. The Bayesian phylogenetic inference resulted in a reliable topology, in agreement with conventional relationships (Simpson and Roger, 2004; Hampl et al., 2009). The results showed a better resolution of classification than the QIIME classification and was confirmed by the intra-structures of 3 clades. However, as mentioned previously, primer selection and the process of PCR might have introduced some biases into the results (Hong et al., 2009). The amplicon products from different 18S rRNA gene regions may also result in different eukaryotic communities (Bricheux et al., 2013). Therefore, the estimation of community compositions using tagged pyrosequencing of 18 rRNA amplicons is still confronting basic technical difficulties. A special issue for studies investigating eukaryotic microbial communities is the copy number variation inherent to rRNA genes of the various eukaryotic genomes. Larger eukaryotic microbes typically have larger genomes and more 18S rRNA gene copies (Prokopowich et al., 2003), resulting in a greater number of amplicons and, subsequently, more pyrosequencing reads. As a result, the number of large eukaryotic microbes within a community could be overestimated. Therefore, the effect of body size on estimate of the community structure should be considered in future studies.

The fungi might be degraders of organic debris at the sea floor. The widespread of the fungal species has been confirmed in several reports (Edgcomb et al., 2011a; Nagahama et al., 2011; Burgaud et al., 2013; Coolen et al., 2013). In this study, the marine sediment samples were also populated mainly by the fungi. However, we did not use the facilities capable to simulate the *in situ* conditions to cultivate the fungi in the sediments. Hence, their activities, particularly at the bottom of the brine, remain unknown to us. On the microbial mat, there was a high percentage of reads derived from annelids and nematodes, which indicated that the mat supported the larvae of these organisms (potentially novel species) living around the cold seeps. The hypersaline and oxygen-depleted bottom of the brine pool near the Thuwal cold seeps was considered to be an extreme environment to eukaryotes (Batang et al., 2012). In the present study, fungi and gregarines were detected in BS9 sediment and were the relatives of the species obtained from similar conditions around the world. The discovery of these species at different geological locations suggested that they distributed more widely than previously imagined in aquatic environments. The spreading of Apicomplexa gregarine parasites is probably coupled to their hosts, potentially enabling them to settle in a new oxygen minimum niche. This is supported by the presence of their 18S amplicons reads in Mat and DS6. For these gregarines, we questioned whether the parasite-like species were metabolically active in the sediment. The sampling site of BS9 was deeper than that of BS8 in the brine pool, and therefore, it had an even lower oxygen level (Table 1). Correspondingly, the percentage of gregarines increased in BS9, which might be evidence of their proliferation in the brine pool. Hence, niche specification was evident for the spreading of species in different aquatic environments, as exemplified by the gregarine parasites in this study.

The interface DBI was typically characterized by a strong chemocline and halocline. This type of brine pool was not always observed around other cold seeps. The eukaryotic species living in bottom water might attempt to colonize the interface within which microbes are nourished due to the strong gradients for chemosynthesis (Wylezich and Jürgens, 2011). In the current study, we provide evidence for the presence of novel Euglenozoa and Alveolata species within the interface. The species present in the long branches did not have similar sequences in other environments, although it is possible that these sequences have not yet been discovered. With this respect, the spreading of these species into the brine pool probably gave rise to endemic speciation. It was reported that some Englenozoa species have episymbiontic bacteria that are critical for the exploration of food sources (Edgcomb et al., 2011b). Re-establishment of the episymbionts on euglenozoans spreading into the brine pool likely occurred during the adaptation. This hypothesis might be tested by fluorescence *in situ* hybridization and scanning electron microscopy. If more layers in the interface were sampled, the change of novel OTU components in abundance at different depths might be revealed. This type of spreading might also be applicable to other environments such as anoxic fjords (Behnke et al., 2006; Orsi et al., 2012b) and the Black Sea (Wylezich and Jürgens, 2011).

The Thuwal seeps created different chemical and salinity gradients and then various microhabitats nearby. As such, it is impossible to compare the eukaryotic communities in all the microhabitats. In the present study, DS3, DS6, and BS8 harbored fungi-dominated communities. In addition, the community of NDW resembled those of the sediments in light of the PCA analysis, likely because it was sampled above the bottom site that approximated DS6. Above the other bottom sites, the communities of water samples differed to some extent from that of NDW while approaching the seeps (unpublished data). Similar findings were obtained for the communities present at different layers of the interface. Here, 18S rRNA gene amplicons could not be generated in samples with a salinity of 65‰, which indicated that the water body in deeper layers and brine sediment were restricted to these eukaryotic microbes. Additional evolutionary and genomics work is required to obtain evidence of the speciation process in the interface.

### Conflict of interest statement

The authors declare that the research was conducted in the absence of any commercial or financial relationships that could be construed as a potential conflict of interest.

## Supplementary material

Figure S1 **Uncultured eukaryotes were similar to the members of clade 2**.

Figure S2 **Long branches formed by free-living Alveolata in DBI**. The 3 OTUs, DBI-298, DBI-164, and DBI-236, represented 77% of the amplicon reads of the 18S rRNA genes from the interface between the bottom water and the brine pool. The maximum-likelihood tree was based on 1000 bootstrap replicates, and bootstrap values >50 are shown.
