# Supplementary material for: Diversity and distribution of eukaryotic microbes in and around a brine pool adjacent to the Thuwal cold seeps in the Red Sea
Source: Front Microbiol. 2014 Feb 4;5:37. doi: 10.3389/fmicb.2014.00037 (PMC3922051; doi:10.3389/fmicb.2014.00037)

Figure S1. Uncultured eukaryotes were similar to the members of clade 2.

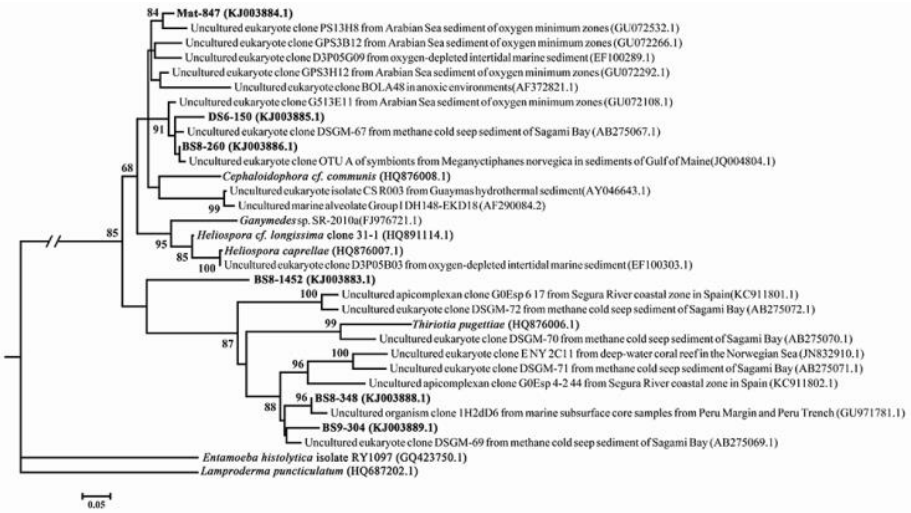

Figure S2. Long branches formed by free-living Alveolata in DBI. The 3 OTUs, DBI-298, DBI-164 and DBI-236, represented 77% of the amplicon reads of the 18S rRNA genes from the interface between the bottom water and the brine pool. The maximum-likelihood tree was based on 1000 bootstrap replicates, and bootstrap values >50 are shown.

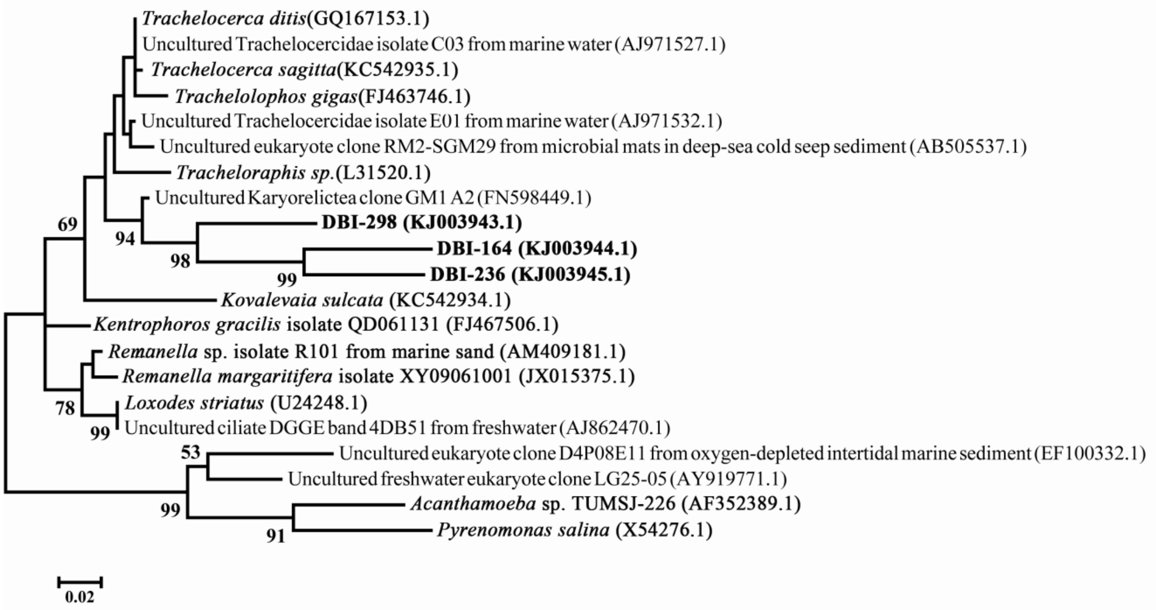

Supplement: Figure S1 — Uncultured eukaryotes were similar to the members of clade 2. [file Presentation1.PDF]
